# Impact of the time of surgical delay on survival in patients with muscle-invasive bladder cancer

**DOI:** 10.3389/fonc.2022.1001843

**Published:** 2022-12-08

**Authors:** Shuaishuai Li, Rui Chen, Ashok Raj, Ning Xue, Fangzheng Zhao, Xihao Shen, Yunpeng Peng, Haitao Zhu

**Affiliations:** ^1^ Department of Urology, The Affiliated Hospital of Xuzhou Medical University, Xuzhou, China; ^2^ Department of Urology, The First Clinical Medical College of Nanjing Medical University, Nanjing, China

**Keywords:** muscle-invasive bladder cancer (MIBC), time of surgical delay, overall survival (OS), cancer-specific survival (CSS), radical cystectomy

## Abstract

**Background and objectives:**

Patients with muscle-invasive bladder cancer (MIBC) often experience a waiting period before radical surgery for numerous reasons; however, the COVID-19 outbreak has exacerbated this problem. Therefore, it is necessary to discuss the impact of the unavoidable time of surgical delay on the outcome of patients with MIBC.

**Methods:**

In all, 165 patients from high-volume centers with pT2-pT3 MIBC, who underwent radical surgery between January 2008 and November 2020, were retrospectively evaluated. Patients’ demographic and pathological information was recorded. Based on the time of surgical delay endured, patients were divided into three groups: long waiting time (> 90 days), intermediate waiting time (30–90 days), and short waiting time (≤ 30 days). Finally, each group’s pathological characteristics and survival rates were compared.

**Results:**

The median time of surgical delay for all patients was 33 days (interquartile range, IQR: 16–67 days). Among the 165 patients, 32 (19.4%) were classified into the long waiting time group, 55 (33.3%) into the intermediate waiting time group, and 78 (47.3%) into the short waiting time group. The median follow-up period for all patients was 48 months (IQR: 23–84 months). The median times of surgical delay in the long, intermediate, and short waiting time groups were 188 days (IQR: 98–367 days), 39 days (IQR: 35–65 days), and 16 days (IQR: 12–22 days), respectively. The 5-year overall survival (OS) rate for all patients was 58.4%, and that in the long, intermediate, and short waiting time groups were 35.7%, 61.3%, and 64.1%, respectively (P = 0.035). The 5-year cancer-specific survival (CSS) rates in the long, intermediate, and short waiting time groups were 38.9%, 61.5%, and 65.0%, respectively (P = 0.042). The multivariate Cox regression analysis identified age, time of surgical delay, pT stage, and lymph node involvement as independent determinants of OS and CSS.

**Conclusion:**

In patients with pT2-pT3 MIBC, the time of surgical delay > 90 days can have a negative impact on survival.

## Introduction

Bladder cancer (BC) is the ninth most common cancer worldwide with approximately 430,000 cases being diagnosed each year and ranks 13^th^ on the list of annual mortality due to cancer (1). In the US alone, there are > 80,000 new cases and 17,000 deaths occurring due to BC each year (2). As per the xyz report, smoking is a major risk factor for BC, with the risk of developing BC being 2.5 times higher in smokers than in nonsmokers (3).

Urothelial carcinoma is the most common pathological subtype of BC. Approximately 25% of patients present with muscle-invasive BC (MIBC) (4). MIBC, with a five-year survival rate, is associated with a poor prognosis and is more prone to metastasis and needs systemic therapy combined with radical surgery and chemotherapy (5).

Radical cystectomy (RC) is considered the standard treatment for MIBC (6). However, lymph node metastasis and postoperative recurrence substantially impact the quality of life in patients with MIBC (7, 8). A study showed that waiting for more than a specific period of time before surgery could negatively impact clinical outcomes (9). Nevertheless, a minimum time of surgical delay is inevitable. Various factors, like the capacity of high-volume centers, patient concerns, preoperative assessment, and treatment of comorbidities before surgery can affect the time of surgical delay in patients with cancer. Furthermore, surgical delays became even more common during the COVID-19 pandemic.

The current findings on the effect of delayed RC on survival outcomes are inconsistent (10). Some studies have shown that a 3-month delay is associated with poor survival outcomes, while others have found that it is safe (11–13). Thus, this study aimed to explore the effect of the time of surgical delay on the survival outcomes of patients with MIBC.

## Patients and methods

In all, 165 patients with pT2-pT3 MIBC who received treatment for RC at a large center in China between January 2008 and November 2020 were included in the study. The inclusion criteria were:(1) Patients with MIBC confirmed by postoperative histopathological findings, with MIBC initially suspected due to hematuria symptoms or physical examination findings.(2) The pathological type was transitional cell carcinoma. The exclusion criteria were: (1) Patients who underwent neoadjuvant therapy, adjuvant chemotherapy, or palliative surgery. (2) Distant metastases. (3) History of multiple (≥ 2) transurethral resection of bladder tumor (TURBT) before radical cystectomy. The demographic and pathological information, including patients’ age, sex, history of smoking, Eastern Cooperative Oncology Group (ECOG) performance status (ECOG PS), body mass index (BMI), T-stage, N-stage, location and size of the tumor, number of lesions, surgical approach and lymphovascular invasion, lymph node dissection (LND), and surgical margin were retrospectively collected. The time of surgical delay was calculated from the pathology reporting of MIBC until the time of RC. In this study, overall survival (OS) was defined as the period from the date of surgery until the date of death. Cancer-specific survival (CSS) was defined as the period from the date of surgery until the date of death attributed to cancer. All patients with incomplete follow-up information or those lost to follow-up were excluded.

First, according to the time of surgical delay, we divided the patients into three groups: long waiting time (> 90 days), intermediate waiting time (30–90 days), and short waiting time (≤ 30 days). Then, each group’s clinicopathological characteristics and survival were compared.

This study was approved by the institutional review board of The Affiliated Hospital of Xuzhou Medical University. All patients provided written informed consent to publish this study. We also invited a senior pathologist to confirm our pathology results. Tumor staging was determined according to the American Joint Committee on Cancer (AJCC) staging system. Tumor classification was based on the 2004 World Health Organization (WHO) grading system.

## Statistical analysis

Continuous variables were compared using the Kruskal–Wallis and one-way ANOVA tests. The OS and CSS were estimated using the Kaplan–Meier survival curve analysis, and the differences among the three groups were compared using the log-rank test. We analyzed the prognostic factors of OS and CSS using univariate and multivariate Cox proportional hazards models. Variables with P < 0.2 in univariate analysis were eventually included in the multivariate analysis. A P value < 0.05 was considered statistically significant. SPSS version 20.0 (IBM Corporation, Armonk, NY, USA) was used for all statistical analyses.

## Results

The study found that the reasons leading to a delay of surgery mainly included the following aspects: (1) Patients had cardiovascular and cerebrovascular accidents and other surgical contraindications. (2) The patient’s physical condition was poor and could not tolerate surgery in a short time. (3) Patients who were found to have BC by examination when receiving surgical treatment in other departments but could not tolerate surgery again in a short time. (4) Some patients with poor economic conditions, lack of understanding of the disease, lack of symptoms in a short time, and refusal of surgery with continuation of conservative treatment. (5) Limited treatment capacity of some large-capacity medical centers in some countries; a large number of patients who need surgery may have to queue up for admission. At the same time, some patients may delay surgery out of fear of COVID-19.

We initially identified 272 patients with MIBC and then excluded those with incomplete clinical data, including the cause of death data. Finally, 165 patients were enrolled in our study. Of these, 85 (51.5%) died: 78 (47.2%) from BC progression and seven (4.3%) from other causes, including three from cardiovascular and cerebrovascular accidents, three from septic shock, and one from acute intestinal obstruction. Among the 165 patients with pT2-pT3 MIBC, 147 were males (89.1%) and 18 were females (10.9%). The median time of surgical delay, age, and BMI of the whole cohort were 33 days (IQR: 16–67 days), 67 years (IQR: 59–74 years), and 23.2 kg/m^2^ (IQR: 21.6–25.2 kg/m^2^), respectively. A total of 140 (84.8%) patients presented with hematuria and 90 (54.5%) had a history of smoking. Although ureterostomy is not a widely accepted means of urinary diversion given the high stenosis/failure rates, a very high rate of ureterostomy (59.4%) for urinary diversion was noted in our study. The demographic information of all patients is presented in **Table 1**.

**Table 1:** Clinical characteristics of patients in three groups.

Varible	All patients (n = 165)	SWT (days) Short <31 (n = 78)	Intermidiate [31,90] (n = 55)	Long >90 (n = 32)	P
Age (yr)	66.1±10.4	68.1±9.6	64.2±11.9	64.7±8.9	0.070
Sex					0.571
Male	147 (89.1)	71 (43.0)	47 (28.5)	29 (17.6)	
Female	18 (10.9)	7 (4.2)	8 (4.8)	3 (1.8)	
Smoke					0.487
Yes	90 (54.5)	46 (27.9)	29 (17.5)	15 (9.1)	
No	75 (45.5)	32 (19.4)	26 (15.8)	17 (10.3)	
ECOG performance status					0.029
0	103 (62.4)	41 (24.8)	37 (22.4)	25 (15.2)	
1	62 (37.6)	37 (22.4)	18 (10.9)	7 (4.2)	
Median Body mass index (kg/m2)	23.6±3.1	24.0±3.2	23.4±3.2	23.1±2.7	0.350
Haematuria					0.070
(+)	140 (84.8)	62 (37.6)	47 (28.5)	31 (18.8)	
(-)	25 (15.2)	16 (9.7)	8 (4.8)	1 (0.6)	
Hydronephrosis					0.834
(+)	50 (30.3)	25 (15.2)	15 (9.1)	10 (6.1)	
(-)	115 (69.7)	53 (32.1)	40 (24.2)	22 (13.3)	
Tumor grade					0.602
High	146 (88.5)	71 (43.0)	47 (28.5)	28 (17.0)	
Low	19 (11.5)	7 (4.2)	8 (4.8)	4 (2.4)	
Tumor size	4.0±1.9	3.8±1.8	4.1±2.0	4.4±1.7	0.324
Amount of lesions					0.398
Single	92 (55.8)	43 (26.1)	34 (20.6)	15 (9.1)	
Mutiple	73 (44.2)	35 (21.2)	21 (12.7)	17 (10.3)	
pT stage					0.475
pT2	116 (70.3)	52 (31.5)	42 (25.5)	22 (13.3)	
pT3	49 (29.7)	26 (15.8)	13 (7.9)	10 (6.1)	
Lymph node involvement					0.751
pN0	134 (81.2)	64 (38.8)	43 (26.1)	27 (16.4)	
pN+	31 (18.8)	14 (8.5)	12 (7.3)	5 (3.0)	
LND					0.182
Yes	155 (93.9)	75 (45.5)	49 (29.7)	31 (18.8)	
No	10 (6.1)	3 (1.8)	6 (3.6)	1 (0.6)	
open or laparoscopy					1.000
Open	57 (34.5)	27 (16.4)	19 (11.5)	11 (6.7)	
Laparoscopy	108 (65.5)	51 (30.9)	36 (21.8)	21 (12.7)	
surgical method					0.461
ureterostomy	98 (59.4)	49 (29.7)	33 (20.0)	16 (9.7)	
ileal orthotopic neobladder	67 (40.6)	29 (17.6)	22 (13.3)	16 (9.7)	
Surgical margin					1.000
Positive	0 (0.0)	0 (0.0)	0 (0.0)	0 (0.0)	
Negative	165 (100.0)	78 (47.3)	55 (33.3)	32 (19.4)	
Infiltrative tumor Architecture					0.214
Yes	147 (89.1)	73 (44.2)	47 (28.5)	27 (16.4)	
No	18 (10.9)	5 (3.0)	8 (4.8)	5 (3.0)	
lymphovascular invasion					
Yes	39 (23.6)	18 (10.9)	16 (9.7)	5 (3.0)	0.360
No	126 (76.4)	60 (36.4)	39 (23.6)	27 (16.4)	

SWT, surgical wait time; ECOG, Eastern Cooperative Oncology Group; pN0, no lymph node involvement; pN+, lymph node involvement; LND, dissection of lymph node.

Of the 165 patients, 32 (19.4%), 55 (33.3%), and 78 (47.3%) were categorized into the long, intermediate, and short waiting time groups, respectively. The median times of surgical delay in the long, intermediate, and short waiting time groups were 188 days (IQR: 98–367 days), 39 days (IQR: 35–65 days), and 16 days (IQR: 12–22 days), respectively. No significant differences were observed among the three groups in terms of age, sex, history of smoking, BMI, grade and size of the tumor, number of lesions, pT stage, lymph node involvement, LND, surgical methods, margin, infiltrative cancer architecture, and lymphovascular invasion.

**Figure 1:**
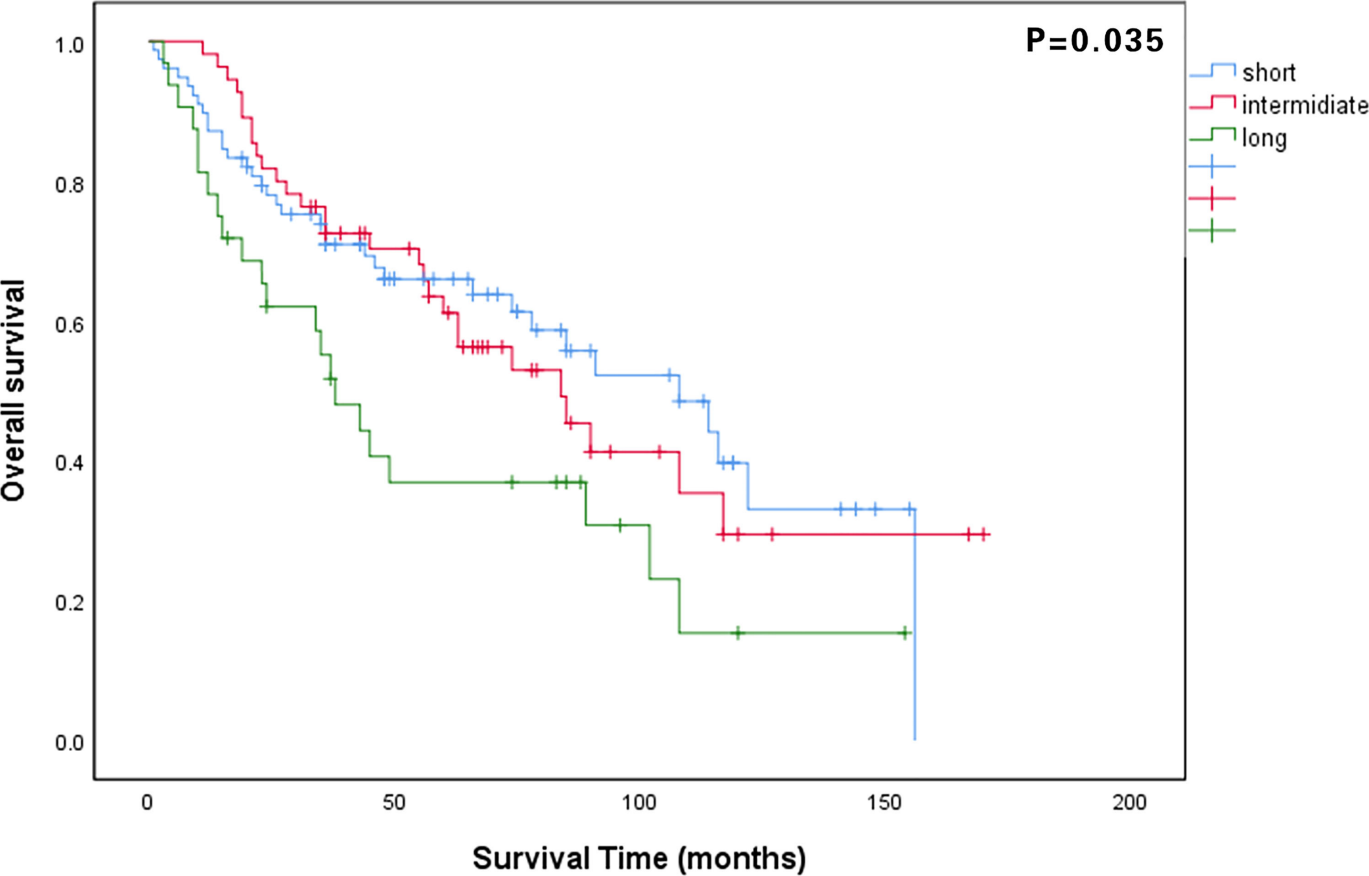
Comparison of overall survival curves among patients in the long, intermediate, and short waiting time groups.

**Figure 2:**
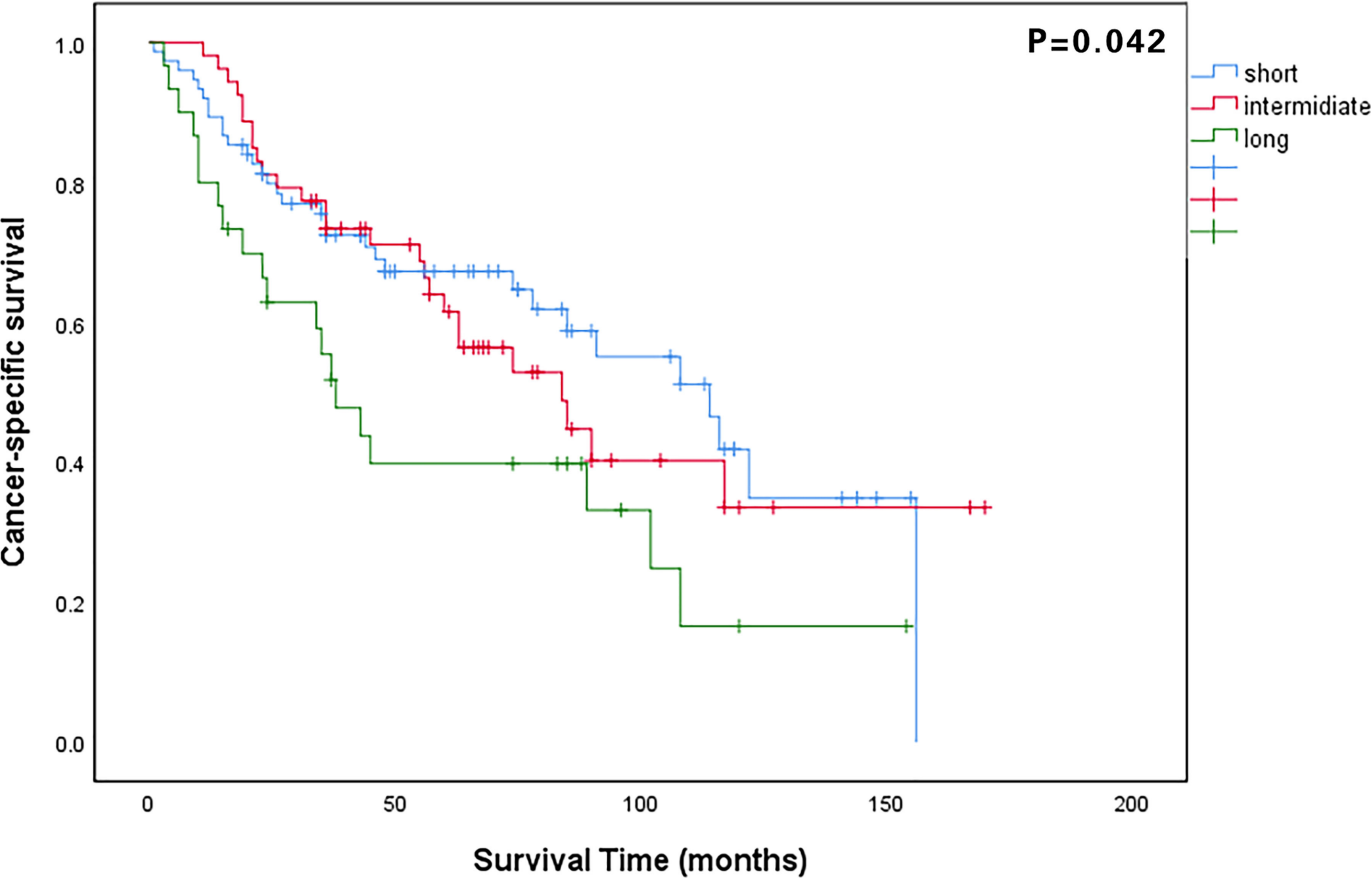
Comparison of cancer-specific survival curves among patients in the long, intermediate, and short waiting time groups.

The median follow-up period for all patients was 48 months (IQR: 23–84 months). The 5-year OS rate of all patients was 58.4%, and that in the long, intermediate, and short waiting time groups were 35.7%, 61.3%, and 64.1%, respectively (P = 0.035). The 5-year CSS rates in the long, intermediate, and short waiting time groups were 38.9%, 61.5%, and 65.0%, respectively (P = 0.042). There was no significant difference in the OS and CSS between the short and intermediate waiting time groups. (**Figures 1**, **2**). However, the OS and CSS of the long waiting time group were obviously shorter than those of the other two groups.

Univariate and multivariate Cox regression analyses identified age, time of surgical delay, pT stage, and lymph node involvement as the determinants of OS and CSS. The number of lesions was found to be an independent risk factor for CSS (**Table 2**).

**Table 2:** Multivariable Cox model for cancer-specific survival and overall survival.

Variables	Cancer-specific survival (CSS)				Overvall survival (OS)			
	Univariate analyses hazard ratios (95% CI)	P	Multivariate analyses hazard ratios (95% CI)	P	Univariate analyses hazard ratios (95% CI)	P	Multivariate analyses hazard ratios (95% CI)	P
Age	1.047 (1.022,1.073)	0.000	1.087 (1.041,1.134)	0.000	1.045 (1.020,1.072)	0.000	1.086 (1.042,1.132)	0.000
Sex	0.853 (0.451,1.611)	0.624			0.798 (0.420,1.514)	0.489		
Wait time	1.356 (1.027,1.790)	0.032	1.732 (1.270,2.362)	0.001	1.375 (1.030,1.837)	0.031	1.672 (1.238,2.259)	0.001
Smoke	1.145 (0.744,1.762)	0.539			1.080 (0.688,1.696)	0.739		
ECOG	1.622 (1.057,2.490)	0.027	0.546 (0.264,1.127)	0.102	1.490 (0.949,2.340)	0.083	0.619 (0.311,1.232)	0.172
Median Body mass index (kg/m2)	1.016 (0.943,1.096)	0.671			0.985 (0.907,1.070)	0.717		
Haematuria	1.352 (0.698,2.620)	0.371			1.267 (0.651,2.465)	0.486		
Hydronephrosis	1.184 (0.742,1.891)	0.479			1.177 (0.722,1.917)	0.513		
Tumor grade	1.498 (0.748,2.997)	0.254			1.413 (0.703,2.840)	0.331		
Tumor size	1.042 (0.934,1.162)	0.465			1.050 (0.937,1.176)	0.399		
Amount of lesions	0.742 (0.481,1.146)	0.179	0.592 (0.362,0.968)	0.037	0.722 (0.458,1.137)	0.160	0.638 (0.407,0.999)	0.050
pT stage	1.743 (1.124,2.702)	0.013	1.802 (1.052,3.085)	0.032	1.921 (1.217,3.031)	0.005	1.626 (0.960,2.753)	0.070
Lymph node involvement	3.142 (1.902,5.191)	0.000	5.487 (2.354,12.788)	0.000	3.242 (1.918,5.480)	0.000	4.892 (2.238,10.693)	0.000
LND	0.582 (0.290,1.168)	0.128	0.570 (0.245,1.324)	0.191	0.504 (0.242,1.053)	0.068	0.773 (0.351,1.700)	0.522
open or laparoscopy	1.090 (0.697,1.703)	0.706			1.197 (0.752,1.904)	0.448		
surgical method	0.479 (0.299,0.769)	0.002	0.906 (0.503,1.633)	0.744	0.456 (0.278,0.748)	0.002	1.010 (0.574,1.778)	0.973
lymphovascular invasion	1.755 (1.104,2.790)	0.017	0.594 (0.265,1.332)	0.206	1.827 (1.133,2.947)	0.014	0.689 (0.323,1.468)	0.334

Univariate and multivariate Cox proportional hazards models were used to analyze prognostic factors for OS and CSS.In univariate analysis, variables with P < 0.2 were finally included in multivariate analysis.

## Discussion

Delays in cancer treatment are often feared to lead to poor outcomes since prolonged times of surgical delay may be associated with the progress or even metastasis of some tumors. Our study found that compared with timely surgery, times of surgical delay > 90 days significantly compromised the survival in those with pT2-pT3 MIBC.The time of surgical delay was found to be an independent risk factor for OS and CSS. This adverse impact affects both OS and CSS by increasing the time from diagnosis to RC.

RC is the standard of care for patients with MIBC stage T2-T4a, N0-Nx, M0, and high-risk and unresponsive NMIBC after TURBT and intravesical therapy alone (10). However, many diagnostic, therapeutic, or surveillance procedures, such as RC, are typically met with delays, which has been the case, especially since the COVID-19 pandemic. Delays in RC can occur for various reasons, including neoadjuvant therapy, necessary preoperative evaluation, serious complications, patients’ requirements, conservative treatment, a busy operative schedule, or a lack of major surgical experience in small centers. In addition, patients’ surgical willingness, economic conditions, contraindications, such as acute cerebral infarction/myocardial infarction and other complications, and other internal factors also affect the time of surgical delay. The final diagnosis cannot be determined from symptoms and imaging examination alone in most patients with MIBC. Patients generally need to undergo an additional cystoscopy to confirm the diagnosis before RC. Oftentimes, patients undergo multiple cystoscopies before being diagnosed with MIBC. Naturally, the waiting time inevitably tends to get prolonged.

In the current research, the association between time of surgical delay and survival was not uniform across different types of cancers. The time of surgical delay reportedly does not affect survival in many kinds of cancers, including lung, pancreas, prostate, and cervical cancers (14–17). In a large retrospective study on 561 patients diagnosed with clinically localized renal cell carcinoma, Qi et al. confirmed that the time of surgical delay of > 3 months may not influence the OS or CSS (18). However, for patients diagnosed with upper tract urothelial carcinoma, Zhao et al. suggested that the time of surgical delay should not be > 3 months (19).

As the most common urological tumor, BC naturally attracted our attention concerning the effect of the time of surgical delay on patient prognosis. Currently, different research institutions have suggested different times of surgical delay for MIBC. In 2006, a Canadian consortium of experts suggested that the maximum waiting time should not exceed 14 days for patients with MIBC (20). In 2009, Gore et al. investigated 441 patients diagnosed with MIBC and concluded that a delay of > 3 months between TURBT and RC was significantly associated with poor specific survival (21), which is consistent with our research. In another study, Kulkarni et al. found that the risk of death increased significantly after a delay of > 40 days among 2,535 patients in whom the median time of surgical delay between TURBT and RC was 50 days (22).

However, some experts believe that a certain amount of waiting time does not affect the survival of patients. Matthew et al. concluded that a reasonable delay from the last TURBT to RC was not independently associated with stage progression and decreased recurrence-free or disease-specific survival (23). Similarly, Bruins et al. indicated that the time of surgical delay > 3 months had no effect on staging and survival (12). Liedberg et al. also found that treatment delays did not influence disease-specific survival (24).

Our findings corroborate those of prior studies in that a 12-week threshold from the time of diagnosis to RC confers an increased risk of mortality, shorter progression-free survival, and an increased incidence of pathologic progression (11, 25–27). As done in these prior studies, we determined the waiting time “cut-off” period as 90 days in the overall population. On the other hand, 47.3% of patients in our study underwent RC within 30 days. It is worth noting that we defined the time of surgical delay as the duration between pathology reporting and RC. However, many patients also experience a considerable waiting period before the pathological diagnosis is confirmed. Such patients, especially those in economically weaker regions, may lack awareness of the disease and ignore symptoms such as hematuria caused by BC. This prolongs the time for diagnosis and creates conditions conducive to disease progression. Given the current medical situation, it is difficult for patients with cancer to arrange surgery immediately after diagnosis. Many patients may develop mental disorders during this difficult period (28, 29). Aside from the time of surgical delay, we also found that age, pT stage, and lymph node involvement were independent risk factors for OS and CSS. Thus, older patients with multiple comorbidities, poor living conditions, and declining physical function are particularly at risk. Higher pT stage and lymph node involvement may represent more advanced cancer and exacerbate the death of the patient.

In the interpretation of our results, several limitations must be pointed out. (1) This study was a single-center, retrospective study with a long follow-up period making selection bias unavoidable. (2) The short waiting time group was more likely to have an ECOG of 0. (3) The time distribution of the long-wait group was relatively varied. (4) Though the incidence of such cases was very low, we did not exclude variant histology (i.e., urothelial + another histological type). All of the above reasons more or less influence our conclusion. Therefore, the effect of the time of surgical delay on patients’ survival needs further confirmatory studies. Finally, due to the small number of patients receiving neoadjuvant chemotherapy and adjuvant chemotherapy, we did not include them in our study. Therefore, our results may not quite reflect a “real-world” clinical scenario.

## Conclusions

The times of surgical delay of > 90 days in patients with pT2-pT3 MIBC can have a negative impact on survival. Age,time of surgical delay, pT stage, and lymph node involvement were all independent factors influencing OS and CSS. For these patients, surgery should be scheduled as soon as possible.

## Data availability statement

The raw data supporting the conclusions of this article will be made available by the authors, without undue reservation.

## Ethics statement

The studies involving human participants were reviewed and approved by The Affiliated Hospital of Xuzhou Medical University. The patients/participants provided their written informed consent to participate in this study.

## Author contributions

Among these authors, SL, RC, AR and NX contributed equally to this work. All authors contributed to the article and approved the submitted version.
